# Pericardial Effusion After Cardiac Surgery: Prevalence, Characteristics, Risk Factors and Management

**DOI:** 10.3390/jcm15083101

**Published:** 2026-04-18

**Authors:** Gaia Cattadori, Anna Picozzi, Elena Tagliabue, Giovanna Elsa Ute Muti Schuenemann, Tiziana Staine, Roberta Chiodelli, Anna Scaglione, Barbara Baronio, Silvia Di Marco, Claudio Anzà

**Affiliations:** 1 Cardiovascular Section, Department of Clinical Sciences and Community Health, University of Milan, 20122 Milan, Italy; 2 IRCCS Multimedica, 20099 Milan, Italy; anna.picozzi@multimedica.it (A.P.); elena.tagliabue@multimedica.it (E.T.); giovanna.muti@multimedica.it (G.E.U.M.S.); tiziana.staine@multimedica.it (T.S.); roberta.chiodelli@multimedica.it (R.C.); anna.scaglione@multimedica.it (A.S.); barbara.baronio@multimedica.it (B.B.); silvia.dimarco@multimedica.it (S.D.M.); claudio.anza@multimedica.it (C.A.); 3 Department of Emergency, Fondazione IRCSS Ca’ Granda, Ospedale Maggiore Policlinico, 20122 Milan, Italy

**Keywords:** pericardial effusion, cardiac surgery, cardiac tamponade

## Abstract

**Background/Objectives:** Pericardial effusion (PEf) is a frequent finding after cardiac surgery. Progression to cardiac tamponade (CT) is a rare but life-threatening complication. Current evidence remains limited due to insufficient data on prevalence, progression predictors and management strategies. **Methods:** We retrospectively analyzed anamnestic, clinical, laboratory, echocardiographic and therapeutic data from 2662 patients (74 ± 11 years) admitted to the Cardiac Rehabilitation ward between 2022 and 2024. **Results:** Among 2152 (81%) cardiac surgery patients, 382 (18%) developed PEf: 58% mild, 38% moderate, and 4% severe. Patients developing PEf tended to be younger and more frequently male. In addition, PEf development was seen more commonly after aortic and combined surgeries. All patients with severe PEf or CT had undergone surgery via sternotomy, whereas minithoracotomy was inversely associated with PEf severity. Postoperative complications occurred in 92% of PEf patients, mainly due to arrhythmia, hemodynamic deterioration, or heart failure. Overall outcome was favourable in 98% of patients. CT occurred in eight patients (2%). Anticoagulation therapy was more frequent among patients who developed PEf or CT. Preventive colchicine was prescribed in only 16% of cases. No PEf-specific therapy was administered in 56% of PEf patients, while corticosteroids and nonsteroidal anti-inflammatory drugs were used in 28% and 8% of cases, respectively, without surgical wound complications. No PEf recurrences were observed during follow up (517 ± 424 days). **Conclusions:** PEf is a common complication after cardiac surgery, more frequently in young males, usually of mild or moderate severity. The majority of these cases resolve using either a conservative or pharmacological approach, predominantly via corticosteroids. Patients undergoing aortic surgery, experiencing postoperative complications (especially arrhythmias), and receiving anticoagulation therapy were associated with severe PEf or CT. Despite guideline recommendations, colchicine remains markedly underutilized.

## 1. Introduction

Pericardial effusion (PEf) is a common finding after cardiac surgery [1,2,3,4]. Early postoperative PEf, defined as occurring within the first 7–10 days, is relatively frequent and generally attributed to surgical bleeding or mechanical consequences of the procedure. In contrast, late PEf (>7 days after surgery) is, in most cases, part of “postpericardiotomy syndrome (PPS),” a clinical condition characterized by worsening or new-onset pericardial and/or pleural effusion, pericardial rubs, chest pain with or without dyspnea, fever, and elevated inflammatory markers [4,5,6,7].

Resolution of PEf is often spontaneous [4,5]; however, progression to cardiac tamponade (CT) represents a life-threatening emergency [8,9]. The 2025 European Society of Cardiology (ESC) Guidelines for the treatment of myocarditis and pericarditis [6] confirm an approach to PEf/PPS that largely mirrors the management of acute pericarditis [6,10], owing to the limited data specifically addressing postoperative PPS or PEf. Identifying patient characteristics associated with postoperative PEf and predictors of progression to CT is therefore crucial to optimizing treatment and management strategies and prioritizing clinical and echocardiographic surveillance, particularly in the cardiac rehabilitation (CR) setting.

The aim of this study was to retrospectively describe a large cohort of patients admitted to a CR setting after cardiac surgery in order to assess PEf prevalence, patient characteristics, clinical course, and the pre-, intra-, and postoperative features associated with PEf severity and/or progression to CT, with the ultimate goal of defining optimal management and therapeutic strategies.

## 2. Materials and Methods

### 2.1. Study Population

We retrospectively described a cohort of 2662 patients (M/F 1775/887, mean age 74 ± 11) who were hospitalized in the CR Units of San Giuseppe Hospital and Santa Maria Rehabilitation Centre, Research and Clinical Centre, Multimedica, Italy, between January 2022 and April 2024.

### 2.2. Anamnestic and Clinical Data

Data were collected from stored electronic medical records (Normadec Srl, Saronno, Italy). Information on age, sex, type of cardiac surgery and length of hospitalization (including both the cardiac surgery and CR stay) was obtained for the entire postoperative cohort. For patients with PEf, anamnestic, surgical and post-surgery data were collected, including comprehensive echocardiographic findings at CR admission and therapeutic strategies adopted during CR stay.

### 2.3. Laboratory Data

Routine laboratory blood samples obtained at the time of echocardiographic evaluation upon CR admission were collected for PEf patients.

### 2.4. Echocardiographic Evaluations

All postoperative patients underwent transthoracic echocardiogram (TTE) (Philips Affiniti 70 ultrasound system) at CR admission, at discharge, and before urgent transfer. For the purposes of this study, echocardiographic data were analyzed for all patients who developed PEf. Examinations were performed using standard echocardiographic techniques, and PEf was assessed by the end-diastolic distance of the echo-free space between the epicardium and parietal pericardium. According to the most recent ESC guidelines for the diagnosis and management of pericardial diseases [6], we classified PEf size as mild (<10 mm), moderate (10–20 mm) or large (>20 mm) and PEf distribution as circumferential or loculated. The diagnosis of CT was based on clinical findings and standard echocardiographic and Doppler criteria, in accordance with current guidelines [6,9], as there is a >25% to 30% decrease in mitral inflow velocity and a >40% to 60% increase in tricuspid velocity in the first beat after inspiration, and the opposite is true during expiration.

#### Therapy

The PEf-targeted anti-inflammatory drugs used in this study included colchicine (0.5 to 1 mg/day), nonsteroidal anti-inflammatory drugs (NSAIDs) (600 mg ibuprofen every 8 h or 25 to 50 mg indomethacin every 8 h, maintained for 1 or 2 weeks then tapered) and corticosteroids (0.2 to 0.5 mg/kg/day prednisone for 1 or 2 weeks then slowly tapered) [6].

### 2.5. Patient Follow-Up

In-hospital follow-up ended at discharge or urgent transfer. TTE and therapy data were collected at this time as well.

Out-of-hospital follow-up ended with the last clinical evaluation collected by regional Social and healthCare Information System, via Multimedica electronic system or through a direct phone call. Data on deaths, cardiovascular hospitalizations (including specific information, if available, such as heart failure, redo procedures, prosthetic valve infection, etc.), and other medical conditions were collected.

### 2.6. Data Management

Data were analyzed for the entire PEf population, and patients were grouped according to PEf severity. Patients transferred for CT were separately analyzed.

The research protocol was approved by the relevant institutional review boards and ethics committees. Informed consent was obtained from all participants (study number: 4240; trial ID: 4656).

### 2.7. Statistical Methods

Categorical variables were presented as frequency and percentage, and between-group comparison was conducted using the chi-square or Fisher’s exact test, as appropriate. Continuous variables were summarized as means ± standard deviation (SD) and tested for normality by the Kolmogorov–Smirnov test. Since variables were not normally distributed, the Kruskal–Wallis test was used to compare variables between the three groups. A *p* < 0.05 was used to define statistical significance. All analyses were performed using SAS software v.9.4 (SAS Institute Inc., Cary, NC, USA).

## 3. Results

**Figure 1:**
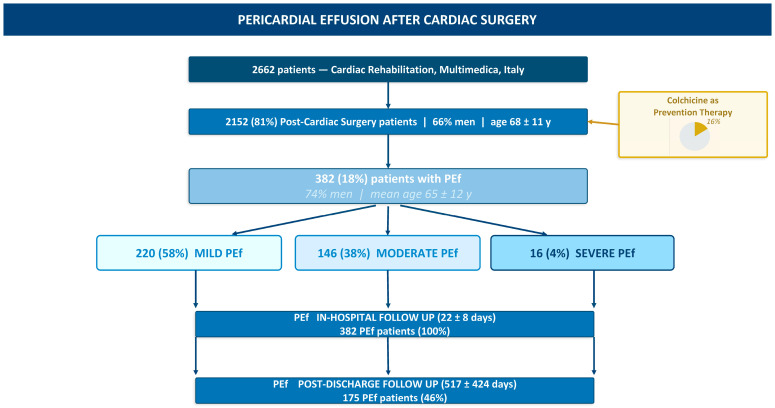
Flowchart of the observational study.

Among the 2662 patients hospitalized in CR Units of the Research and Clinical Centre Multimedica, Italy, between January 2022 and April 2024, 2152 were post-cardiac surgery patients (81% of the total population; 66% men; mean age: 68 ± 11 years). PEf was detected in 382 patients (14% of total population and 18% of the post-cardiac surgery population; 74% men; mean age: 66 ± 11 years). Patients who developed PEf tended to be younger and more frequently male compared to patients without PEf. PEf severity was classified as mild in 220 patients (58%), moderate in 146 (38%), and severe in 16 (4%) (Figure 1) (Table 1).

Among patients who did not develop PEf, 26% underwent coronary artery bypass graft (CABG) surgery, 51% underwent valve surgery, 4% underwent aortic surgery, 6% underwent combined valve and aortic surgery, 10% underwent combined CABG and valve surgery, and 3% underwent other types of cardiac surgery. Among PEf patients, 85 (22%) underwent CABG, 162 (42%) underwent valve surgery, 26 (7%) underwent aortic surgery, 40 (10%) underwent combined valve and aortic surgery, 63 (16%) underwent combined CABG and valve surgery, and 6 (2%) underwent other cardiac procedures. Among all patients enrolled in this study, no ice or chilled solutions were used in the pericardium during surgery. Aortic and combined surgery were more frequently associated with PEf development (Table 1). Notably, aortic involvement was present in 88% of patients transferred due to CT (Table 2). Redo surgery, atrial fibrillation ablation, left atrial appendage occlusion, mechanical valve replacement, and urgent surgery did not differ between PEf severity levels. Finally, 100% of patients who developed severe PEf or CT were treated via a sternotomic approach, while minithoracotomy was inversely associated with PEf severity (Table 2).

Common pre-surgical characteristics of PEf patients (Table 3) included a high body mass index (BMI), seen in 47% of patients, and a mean left ventricular ejection fraction (LVEF) of 58% ± 10, with values below the normal range in 15% of total PEf patients. Notably, no patients with LVEF < 40% developed severe PEf or progressed to CT. Arterial hypertension was frequently reported in the medical history of PEf patients. No anamnestic variables differed based on PEf severity.

Post-surgical course was characterized by at least one complication in 92% of PEf patients, with arrhythmic and haemodynamic/heart failure (HF) complications being the most frequent (Table 4). Postoperative atrial fibrillation (POAF) occurred in 54% of PEf patients. PEf was detected before CR admission (early PEf) in only 54 patients (14%) and less frequently among those undergoing CABG. Half of CT cases occurred in patients with early PEf. All CT patients experienced at least two postoperative complications, including arrhythmic events in 88% of cases (63% POAF).

Despite this, the length of postoperative hospitalization was not significantly prolonged in PEf patients compared with those without PEf (Table 1).

PPS criteria, blood chemistry and TTE data of PEf patients were collected at CR admission, which tended to be after 13 ± 11 days post-surgery. Diagnostic criteria for PPS were present in 70% of PEf patients but were less frequent in those with severe PEf, who also exhibited lower C-reactive protein (CRP) levels.

TTE data found in PEf patients were: left ventricular (LV) hypertrophy, normal LV volumes, normal LVEF, and left atrial enlargement, without significant valvular disease. These were also observed in patients with CT. Left-sided loculated PEf was more frequently present in subjects with severe PEf, whereas circumferential PEf, observed in 66% of patients, was more frequent in mild PEf.

**Figure 2:**
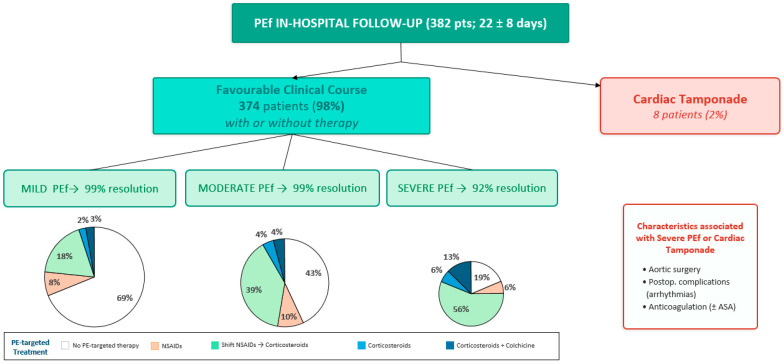
PEf in-hospital follow-up.

**Figure 3:**
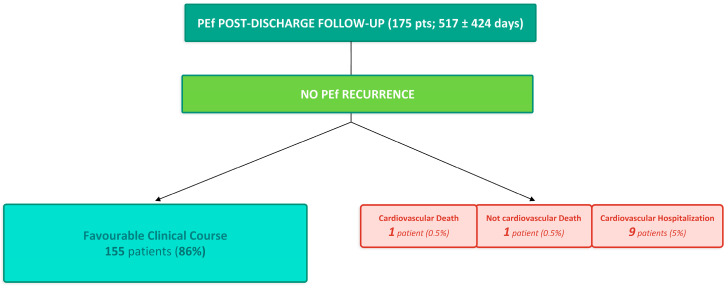
PEf post-discharge follow-up.

The evolution of PEf is reported in Table 5 and Figure 2 and Figure 3. Overall, 98% of PEf patients (374/382) showed a rapid and favourable in-hospital course, with improvement or resolution of PEf at TTE performed before discharge. Specifically, improvement was observed in 99% of patients with mild PEf (217), 99% with moderate PEf (145), and 75% with severe PEf (12), after a mean CR stay of 22 days. CT requiring urgent transfer occurred in 8 patients (0.4% of total post-surgical population and 2% of total PEf patients). CT was detected at the first TTE in three symptomatic patients. These had an evolution from mild effusion in three patients, from moderate PEf in one patient, and from severe PEf in one patient. After hospital discharge (mean follow-up: 517 ± 424 days; *n* = 175), two deaths occurred, both in patients with mild PEf who did not receive PEf-targeted therapy (one due to an accidental cause and one due to cardiocirculatory arrest secondary to HF). Nine patients were re-hospitalized due to cardiovascular causes (one REDO surgery for prosthetic valve infection, four HF, two arrhythmic disorders, and two vascular conditions of unspecified etiology). Of these, five had mild PEf and four had moderate PEf at first TTE; no PEf-targeted therapy was initiated in six patients, whereas three received corticosteroids. Notably, no recurrent PEf events occurred during follow-up after hospital discharge.

Treatment strategies for PEf patients are reported in Table 6 and illustrated in Figure 2. Anticoagulation therapy, with or without acetylsalicic acid (ASA), was more frequently administered in patients who developed severe PEf or CT (88% and 75%, respectively). For PEf prevention, colchicine was prescribed in only 62 patients (16% of total PEf population). PEf-targeted therapy was not initiated in 214 PEf patients (56%), including 151 (69%) with mild PEf and 63 (43%) with moderate PEf. Corticosteroids were administered to 106 PEf patients (28%): 40 with mild PEf (18%), 57 with moderate PEf (39%), and 9 with severe PEf (56%). NSAIDs were administered in 32 patients (8%): 17 with mild PEf (8%), 14 with moderate PEf (10%), and 1 with severe PEf (6%). Early PEf patients were more frequently treated with corticosteroids (52%), whereas a conservative approach without PEf-targeted therapy was more commonly applied in late PEf patients. No surgical wound complications were observed in patients treated with corticosteroids.

Table 7 summarizes data from patients transferred due to CT. Urgent transfer due to CT occurred at the time of initial PEf diagnosis due to the unfavourable progression of mild PEf in three patients (without prior PEf-target treatment), due to the worsening of moderate PEf in one patient despite 10 days of corticosteroids therapy, and due to the worsening of severe PEf in one patient despite 3 days of combined corticosteroid and colchicine therapy. All CT patients underwent sternotomy and had a post-surgical course characterized by at least two complications. All but one CT patients had cardiac surgery involving the aorta, 50% had early PEf and all received anticoagulation therapy (three with anticoagulation alone and three with anticoagulation plus ASA), except for two patients treated with ASA in association with enoxaparin at antithrombotic dosage.

## 4. Discussion

This study represents the largest contemporary retrospective observational study investigating the prevalence, characteristics and management of post-cardiac surgery PEf in patients admitted to the CR wards.

Our data confirms the high prevalence of PEf in patients admitted to CR following cardiac surgery, more frequently of mild or moderate severity, and demonstrates a generally rapid and favourable PEf evolution with rare progression to CT. This occurred regardless of whether patients were treated with corticosteroids, NSAIDs, or managed conservatively. Patients who developed PEf tended to be younger and more frequently male compared to patients without PEf. Aortic surgery involvement, the presence of postoperative complications, particularly arrhythmias, and anticoagulation therapy, with or without ASA, were seen to be associated with severe PEf manifestation and progression to CT. These findings support the need for closer surveillance and tailored preventive strategies in this patient population. Moreover, despite being recommended by current guidelines for PEf in the context of post-PPS, colchicine remains markedly underutilized and should be implemented more consistently. Finally, no recurrent PEf events were documented during follow up.

### 4.1. Prevalence of PEf and CT

Our data confirm that PEf is common in patients admitted to CR wards after cardiac surgery, with a prevalence of 18%. Similarly, Meurin et al. [9] reported PEf in 22% of postoperative patients, and Imazio et al. observed PEf in 15% of patients enrolled in the COPPS trial [11,12]. In contrast, earlier studies described substantially higher incidences ranging from 53 to 85%, largely reflecting the earlier timing of echocardiographic assessments in these cohorts [1,2,3,9].

Despite the expansion of surgical indications to an older and more comorbid population, our findings also confirm a low prevalence of postoperative CT in this population, in line with previous reports showing rates between 0.8 and 4% [1,2,9]. By contrast, Van Osch et al. [13], in a sub-analysis of a single-centre cohort of patients undergoing non-emergent valve surgery, demonstrated a 10-fold increased risk of reoperation for CT in 2–months in patients with PPS detected on postoperative days 4 to 6. These data nonetheless reinforce the need for vigilant monitoring of early PPS in the CR setting.

### 4.2. Characteristics Associated with PEf Severity and Predictive of Progression to CT

#### 4.2.1. Anamnestic Data

Patients who developed PEf in CR setting were younger than postoperative patients without PEf, in line with previous observations [14]. These were predominantly male, in contrast to what has been reported in patients with PPS or non-idiopathic pericarditis—conditions more frequently associated with female sex [15]. A more detailed analysis of the anamnestic data of PEf identifies a characteristic clinical profile of individuals associated with PEf development: younger age, male sex, elevated BMI, preserved LVEF, and history of arterial hypertension. Interestingly, and contrary to expectations, no patients with preoperative LVEF < 40% developed severe PE or progressed to CT.

#### 4.2.2. Type of Surgery

Meurin et al. [9] and Pepi et al. [3] previously reported a significantly higher incidence of PEf after CABG compared to valvular surgery. More recently, Kuvin et al. [8], Letho et al. [14] and Pompilio et al. [16] identified cardiac operations other than isolated CABG as potential risk factors for postoperative PEf, likely related to pericardial closure and the frequent need for anticoagulantion therapy. Consistent with these observations, our data demonstrate a strong association between aortic surgery involvement and both PEf development and adverse progression to CT in patients admitted to the CR setting. Notably, we also report a clinically relevant finding: a sternotomy surgical approach was performed in all patients with severe PEf and CT.

#### 4.2.3. Post Surgery Complications (Number and Type)

Patients with a complicated postoperative course, particularly those experiencing episodes of postoperative atrial fibrillation (POAF), should be closely monitored for the development of PEf in CR setting, as previously reported [7,17]. HF and early PEf appear to be more frequently associated with progression to CT.

#### 4.2.4. Blood Samples Data and PPS

Lower CRP levels observed in patients with severe PEf, together with the lower prevalence of PPS diagnostic criteria in this group, support the idea that PEf occurring in the context of PPS represents a distinct nosological entity, generally associated with a more favourable clinical course [18].

#### 4.2.5. Not PEf-Targeted Therapy

Considerable debate exists regarding the role of anticoagulant therapy as a causal factor in the development of PEf after cardiac surgery. In our study, most patients with severe PEf or CT were receiving anticoagulants, in line with previous reports demonstrating a higher incidence of late CT among anticoagulated patients [2,9]. These findings underscore the need for close clinical and echocardiographic monitoring in this subgroup.

#### 4.2.6. PEf Management/Treatment

Several pharmacological strategies, including aspirin, methylprednisolone, dexamethasone, and colchicine, have been evaluated for the prevention and treatment of postoperative PEf [6,11,12,19,20,21,22]. However, with the exception of colchicine for primary prevention [19,23,24,25], currently available evidence does not support a standardized, evidence-based clinical and pharmacological management of postoperative PEf.

Colchicine, owing to its anti-inflammatory properties, has demonstrated efficacy across a broad spectrum of cardiovascular conditions, most notably in the treatment of acute and recurrent pericarditis [26,27,28]. Based on its proven ability to reduce the risk of PEf in the PPS setting, colchicine is recommended for primary prevention of post cardiac surgery PEf/PPS [19,23,24,25]. Nevertheless, our data indicates that colchicine remains markedly underutilized in post-cardiac surgical patients, highlighting the need to reinforce its use more consistently in clinical practice.

Given the frequent spontaneous resolution of PEf, the 2025 ESC Guidelines for the management of myocarditis and pericarditis, recommend limiting treatment in the context of PPS to moderate or severe effusions with signs of inflammation. This recommendation aligns with previous studies showing that the incidence of late CT increases significantly with PEf severity [3,9]. Meurin et al. [9] validated a postoperative severity classification, reporting that no patients with a PEf grade of <2 (loculated effusion: 10–14 mm; circumferential effusion < 10 mm) progressed to CT. By contrast, in our study, three cases of CT were identified at the first echocardiographic evaluation, three evolved from mild PEf, one from moderate, and one from severe. Although the small number of CT cases in our cohort [8], as well as in previous reports (15 in Pepi et al. [3] and 15 in Meurin et al. [9]), precludes definitive recommendations, our findings suggest that CT can also develop in patients with mild PEf. This observation challenges prior recommendations to restrict clinical and echocardiographic surveillance to patients with loculated PEf > 10–14 mm or circumferential PEf > 10 mm.

Regarding treatment, the 2025 ESC Guidelines [6] reaffirm the lack of specific evidence for post-surgical PEf in the context of PPS and recommend an approach analogous to that used for acute pericarditis, based on empirical anti-inflammatory therapy. High-dose aspirin or NSAIDs, combined with proton pump inhibitors, are considered first-line treatments to control symptoms and reduce recurrences. In post-cardiac surgery patients, particularly those with PPS or receiving concomitant therapies such as oral anticoagulants, corticosteroids are also recommended [29,30,31]. In our cohort, the predominant strategy for mild PEf was conservative management without specific treatment. This supported previous suggestions [32] that careful clinical and echocardiographic monitoring represent a valid alternative to PEf-targeted treatment, especially in patients at high risk of bleeding. Conversely, in our cohort, most patients with moderate PEf and all patients with severe PEf were treated with corticosteroids, alone or in combination with colchicine. Corticosteroids were often preferred over NSAIDs due to ongoing anticoagulant therapy. This strategy was associated with a high rate of favourable clinical outcomes, with improvement or resolution of PEf at pre-discharge TTE in 98% of PEf patients, without any observed surgical wound complications in those receiving corticosteroids.

#### 4.2.7. Follow Up

Letho et al. [33] conducted a large-scale nationwide epidemiological study evaluating PPS and long-term mortality, with a median follow up of 9 years. Only PPS episodes severe enough to require hospital admission or to contribute to death were included. This study demonstrated, for the first time, that the development of PPS requiring invasive interventions was associated with increased mortality after cardiac or ascending aortic surgery. In contrast, in our cohort, no recurrent PEf events were observed during follow up, and only two deaths occurred, neither of which were related to pericardial complications. Direct comparison with the study by Letho et al. is limited by the shorter post-discharge follow-up in our study and by differences in patient selection, as our population was not restricted to clinically severe cases. Further research is warranted to better clarify the long-term prognostic implications of PPS/PEf across the full clinical spectrum. Nevertheless, within the limits of our follow-up duration, our management strategy appeared effective without recurrent PEf event and was not associated with treatment-related complications, including surgical wound issues in patients receiving corticosteroids.

#### 4.2.8. Limitations of the Study

The limitations of our study are primarily attributable to the retrospective and observational nature of this project. First of all, it is imperative to further underscore the study environment of our research. Our findings may not fully represent the entire spectrum of PEf, as the echocardiographic assessment was conducted in a CR population rather than in the immediate postoperative surgical setting. Moreover, even if the descriptive data on colchicine, corticosteroids, NSAIDs, and drainage strategies are valuable, any comparative conclusions regarding treatment effectiveness or safety was limited due to the lack of standardization in the treatment process.

## 5. Conclusions

Our retrospective observational study highlights several clinically relevant findings regarding PEf after cardiac surgery in patients admitted to the CR setting:

(1) PEf is highly prevalent, mainly in young male patients;

(2) the clinical course is generally favourable, whether managed conservatively or treated with corticosteroids (often preferred) or NSAIDs.

(3) patients undergoing aortic surgery, experiencing postoperative complications (especially arrhythmias), and receiving anticoagulation therapy were associated with severe PEf manifestation and progression to CT; a sternotomy surgical approach was performed in all patients with severe PEf and CT;

(4) the use of colchicine for PEf prevention remains underutilized and should be reinforced in accordance with current guideline recommendations.

## Figures and Tables

**Table 1:** Characteristics of the entire cardiac surgery study population (*n* = 2152, 81% of the total population of 2662 patients) grouped according to pericardial effusion presence (PEf, *n* = 382, 18% of the total cardiac surgery patients) or PEf absence (no PEf, *n* = 1770, 82% of the total cardiac surgery patients).

	No PEf (*n* = 1770)	PEf (*n* = 382)	*p*
Demographics			
Age (years)	69 ± 11	66 ± 11	<0.0001
Sex (male)	1150 (65%)	281 (74%)	<0.005
Type of Surgery			
CABG	458 (26%)	85 (22%)	Ns
VALVE	894 (51%)	162 (42%)	0.0041
AORTIC	73 (4%)	26 (7%)	0.0233
VALVE/AORTIC	109 (6%)	40 (10%)	0.0026
COMBINED	175 (10%)	63 (16%)	0.0002
Other	56 (3%)	6 (2%)	Ns
Hospital Stay			
Overall hospital stay (days)	32 ± 8	32 ± 13	Ns
Cardiac surgery stay (days)	9 ± 7	9 ± 9	Ns
Rehabilitation stay (days)	22 ± 10	22 ± 8	Ns

CABG = coronary artery bypass graft; VALVE = aortic or mitral valve surgery; AORTIC = aortic surgery; COMBINED = CABG + VALVE/AORTIC; Ns = not significant.

**Table 2:** Surgery data of PE patients grouped according to severity and cardiac tamponade.

	PEf Total *n* = 382	Mild PEf *n* = 220 (58%)	Moderate PEf *n* = 146 (38%)	Severe PEf *n* = 16 (4%)	Cardiac Tamponade *n* = 8 (2%)	*p*
Surgery Data						
CABG	85 (22%)	54 (25%)	29 (20%)	2 (13%)	1 (13%)	Ns
Valve	162 (42%)	92 (42%)	62 (42%)	8 (50%)	2 (25%)	Ns
Aortic	26 (7%)	10 (5%)	15 (10%)	1 (6%)	1 (13%)	Ns
Valve/Aortic	40 (10%)	19 (9%)	19 (13%)	2 (13%)	3 (38%)	Ns
Combined	63 (16%)	42 (19%)	18 (12%)	3 (19%)	1 (13%)	Ns
Other	6 (2%)	3 (1%)	3 (2%)	0 (0%)	0	Ns

Aortic surgery involvement	152 (59%)	86 (58%)	58 (59%)	8 (73%)	7 (88%)	Ns
Mitral surgery involvement	106 (41%)	62 (42%)	41 (41%)	3 (27%)	0	Ns
Special Procedures						
REDO	24 (6%)	12 (5%)	12 (8%)	0	1 (13%)	Ns
Atrial fibrillation ablation	14 (4%)	7 (3%)	7 (5%)	0	0	Ns
Left auricle occlusion	35 (9%)	15 (7%)	17 (12%)	3 (19%)	0	Ns
Mechanic valve	17 (4%)	9 (4%)	7 (5%)	1 (6%)	0	Ns
Urgency	18 (5%)	8 (4%)	10 (7%)	0	0	Ns
Surgical Approach						
Sternotomy	335 (88%)	189 (86%)	130 (89%)	16 (100%)	8 (100%)	Ns
Minithoracotomy	24 (6%)	20 (9%)	4 (3%)	0	0	<0.05
Ministernotomy	23 (6%)	11 (5%)	12 (8%)	0	0	Ns

For legend, see Table 1. Aortic surgery involvement = all surgical procedures involving the aortic valve or the aorta; Ns = not significant.

**Table 3:** Pre-surgery characteristics of PEf patients according to severity and cardiac tamponade.

Pre-Surgery Characteristics	PEf Total *n* = 382	Mild PEf *n* = 220 (58%)	Moderate PEf *n* = 146 (38%)	Severe PEf *n* = 16 (4%)	Cardiac Tamponade *n* = 8 (2%)	*p*
BMI > 25	130 (47%)	71 (46%)	56 (50%)	3 (27%)	1 (33%)	Ns
BMI > 30	44 (16%)	26 (17%)	17 (15%)	1 (9%)	1 (33%)	Ns
LVEF (%)	58 ± 10	58 ± 10	57 ± 10	58 ± 11	61 ± 7	Ns
LVEF 40–50%	56 (24%)	28 (20%)	24 (28%)	4 (36%)	2 (25%)	Ns
LVEF < 40%	13 (6%)	8 (6%)	5 (6%)	0	0	Ns
CAD	74 (19%)	44 (20%)	27 (18%)	3 (19%)	2 (25%)	Ns
Previous Endocarditis	25 (7%)	16 (7%)	8 (5%)	1 (6%)	0	Ns
Previous Pericarditis	2 (1%)	1 (0.5%)	1 (1%)	0	0	Ns
Aortic dissection	11 (3%)	3 (1%)	8 (5%)	0	0	Ns
Arrhythmic problems	67 (18%)	37 (17%)	26 (18%)	4 (25%)	0	Ns
Renal insufficiency	38 (10%)	22 (10%)	16 (11%)	3 (19%)	1 (13%)	Ns
Peripheral vasculopathy	32 (8%)	21 (10%)	10 (7%)	1 (6%)	2 (25%)	Ns
Neurologic problems	38 (10%)	19 (9%)	17 (12%)	2 (13%)	1 (13%)	Ns
Allergies	63 (16%)	41 (19%)	19 (13%)	3 (19%)	1 (13%)	Ns
Diabetes mellitus	69 (18%)	43 (20%)	21 (14%)	5 (31%)	3 (38%)	Ns
Previous smoking	127 (33%)	68 (31%)	50 (34%)	9 (56%)	3 (38%)	Ns
Arterial hypertension	233 (61%)	130 (59%)	94 (64%)	9 (56%)	4 (50%)	Ns

BMI = body mass index; LVEF = left ventricle ejection fraction; CAD = coronary artery disease; Ns = not significant.

**Table 4:** Post cardiac surgery complications, post-pericardiectomy syndrome (PPS) criteria, blood chemistry and echocardiographic data of PEf patients grouped by severity and cardiac tamponade.

Post-Surgery Data	PEf Total *n* = 382	Mild PEf *n* = 220 (58%)	Moderate PEf *n* = 146 (38%)	Severe PEf *n* = 16 (4%)	Cardiac Tamponade *n* = 8 (2%)	*p*
Number of Complications (no. pts)						
No complications	32 (8%)	21 (10%)	10 (7%)	1 (6%)	0 (0%)	Ns
1 complication	80 (21%)	45 (20%)	31 (21%)	4 (25%)	0 (0%)	
2 complications	92 (24%)	56 (25%)	33 (23%)	3 (19%)	2 (25%)	
3 complications	91 (24%)	45 (20%)	44 (30%)	2 (13%)	3 (38%)	
4+ complications	87 (23%)	53 (24%)	28 (19%)	6 (38%)	3 (38%)	
Nature of Complications (no. pts)						
Arrhythmias	224 (59%)	129 (59%)	83 (57%)	12 (75%)	7 (88%)	Ns
POAF	207 (54%)	116 (53%)	81 (55%)	10 (63%)	5 (63%)	Ns
Blood complications	98 (26%)	56 (25%)	39 (27%)	3 (19%)	2 (25%)	Ns
Respiratory complications	125 (33%)	65 (30%)	54 (37%)	6 (38%)	3 (38%)	Ns
Pneumothorax	29 (8%)	14 (6%)	14 (10%)	1 (6%)	0	Ns
Thoracentesis	9 (2%)	6 (3%)	3 (2%)	0 (0%)	0	Ns
Hemodynamic complications	171 (45%)	103 (47%)	62 (42%)	6 (38%)	5 (63%)	Ns
Heart failure	80 (21%)	44 (20%)	33 (23%)	3 (19%)	4 (50%)	Ns
Early PE	54 (14%)	27 (50%)	22 (41%)	5 (9%)	4 (50%)	Ns
Infective complications	117 (31%)	68 (31%)	45 (31%)	4 (25%)	2 (25%)	Ns
Renal complications	42 (11%)	25 (11%)	13 (9%)	4 (25%)	1 (13%)	Ns
Hepatic complications	17 (4%)	8 (4%)	8 (5%)	1 (6%)	1 (13%)	Ns
Wound complications	24 (6%)	13 (6%)	9 (6%)	2 (13%)	0	Ns
Neurologic complications	5 (1%)	3 (1%)	2 (1%)	0 (0%)	0	Ns
Post-Pericardiotomy Syndrome (PPS) Criteria (no. pts)						
PPS criteria	269 (70%)	147 (67%)	113 (77%)	9 (56%)	6 (75%)	<0.05
Hyperpyrexia	109 (29%)	56 (25%)	49 (34%)	4 (25%)	3 (38%)	Ns
Pleural effusion	239 (63%)	134 (61%)	97 (68%)	8 (53%)	6 (86%)	Ns
Blood Chemistry						
Hemoglobin (g/dL)	10.3 ± 1.2	10.4 ± 1.2	10.1 ± 1.2	10.2 ± 1.2	10.2 ± 1.6	Ns
Albuminemia (g/dL)	2.9 ± 0.3	2.9 ± 0.3	2.9 ± 0.4	2.9 ± 0.2	2.8 ± 0.2	Ns
C-reactive protein (mg/L)	7.2 ± 5.1	6.9 ± 5.1	7.8 ± 5.1	4.5 ± 2.2	6.5 ± 3.7	<0.05
Echocardiographic Data						
Septal wall thickness (mm)	12.5 ± 1.8	12.4 ± 1.8	12.5 ± 1.7	13.2 ± 2.4	12.8 ± 1.9	Ns
LV diastolic volume/BSA (mL/m^2^)	52.9 ± 16.4	53.5 ± 16.9	52.2 ± 15.9	52.8 ± 16.9	54 ± 14	Ns
LV ejection fraction (%)	52.7 ± 8.4	52.6 ± 8.6	52.7 ± 7.8	55.2 ± 11.3	52 ± 9	Ns
LV ejection fraction < 40% (no. pts)	13 (6%)	8 (6%)	5 (6%)	0 (0%)	0	Ns
Left atrial enlargement (no. pts)	212 (61%)	113 (57%)	86 (64%)	13 (93%)	6 (86%)	<0.05
E/A	1 ± 0.3	1 ± 0.3	1.1 ± 0.4	1.2 ± 0.3	1.2 ± 0.6	Ns
Mitral regurgitation (1–4 grade)	0.8 ± 0.5	0.8 ± 0.5	0.8 ± 0.5	0.8 ± 0.5	1.1 ± 0.2	Ns
Tricuspid regurgitation (1–4 grade)	0.7 ± 0.6	0.7 ± 0.6	0.7 ± 0.5	0.8 ± 0.5	0.8 ± 0.6	Ns
PE measurements (mm)	13.7 ± 4.8	9.2 ± 1.2	14.7 ± 2.5	26.6 ± 3.5	22.5 ± 8.8	<0.0001
Circumferential (no. pts)	252 (66%)	159 (72%)	85 (58%)	8 (50%)	5 (63%)	<0.01
Loculated—left side (no. pts)	55 (15%)	22 (10%)	26 (18%)	7 (44%)	1 (13%)	<0.001
Loculated—light side (no. pts)	75 (19%)	39 (18%)	35 (24%)	1 (6%)	2 (25%)	Ns

POAF = post-operative atrial fibrillation; LV = left ventricle; Ns = not significant.

**Table 5:** PEf Evolution (in-hospital and post-discharge follow-up).

	PEf Total *n* = 382	Mild PEf *n* = 220 (58%)	Moderate PEf *n* = 146 (38%)	Severe PEf *n* = 16 (4%)
In-Hospital Follow-Up (22 ± 8 days, 382 pts)				
Discharge (no. pts)	374 (98%)	217 (99%)	145 (99%)	12 (75%)
Urgent transfer due to CT (no. pts)	8 (2%)	3 (1%)	1 (1%) *	4 (25%) °
Post-Discharge Follow-Up (517 ± 424 days, 175 pts)				
Deaths (no. pts)	2	2	0	0
CV Hospitalizations (no. pts)	9	5	4	0
Heart failure	4	3	1	0
REDO (prosthetic valve infection)	1	1	0	0
Vascular problems	2	1	1	0
Arrhythmia problems	2	0	2	0

CT = cardiac tamponade; CV = cardiovascular; * after 3 days of corticosteroid therapy; ° urgent transfers immediately after PEf detection and after 10 days of corticosteroids + colchicine.

**Table 6:** Therapy data of PEf patients grouped according to severity and cardiac tamponade.

Therapy	PEf Total *n* = 382	Mild PEf *n* = 220 (58%)	Moderate PEf *n* = 146 (38%)	Severe PEf *n* = 16 (4%)	Cardiac Tamponade *n* = 8 (2%)	*p*
General Medications (no. pts)						
Beta-blockers	330 (86%)	195 (89%)	123 (84%)	12 (75%)	6 (75%)	Ns
Diuretics	266 (70%)	158 (72%)	98 (67%)	10 (63%)	6 (75%)	Ns
ACE-inhibitors/sartans	126 (33%)	72 (33%)	49 (34%)	5 (31%)	3 (38%)	Ns
Mineralocorticoid receptor antagonists	207 (54%)	129 (58%)	73 (50%)	5 (31%)	3 (38%)	Ns
Amiodarone	147 (38%)	81 (37%)	59 (40%)	7 (44%)	5 (63%)	Ns
Antibiotics	147 (38%)	85 (39%)	58 (40%)	4 (25%)	3 (38%)	Ns
Calcium antagonist	22 (6%)	11 (5%)	9 (6%)	2 (13%)	0	Ns
Enoxaparin (antithrombotic dose)	8 (2%)	5 (2%)	2 (1%)	1 (6%)	3 (38%)	Ns
Anticoagulants (oral or subcutaneous)	225 (59%)	131 (60%)	80 (55%)	14 (88%)	6 (75%)	<0.05
Acetylsalicic acid	225 (59%)	138 (63%)	79 (54%)	8 (50%)	5 (63%)	Ns
Anticoagulants + acetylsalicic acid	87 (23%)	57 (26%)	24 (16%)	6 (38%)	3 (38%)	<0.05
Dual antiplatelet therapy	21 (5%)	12 (5%)	8 (6%)	1 (6%)	0	Ns
PEf Prevention Therapy						
Colchicine (pre-treatment)	62 (16%)	20 (9%)	33 (23%)	9 (56%)	1 (13%)	<0.0001
PEf Treatment Therapy						
NSAIDs	32 (8%)	17 (8%)	14 (10%)	1 (6%)	0	Ns
Corticosteroids	106 (28%)	40 (18%)	57 (39%)	9 (56%)	2 (13%)	<0.0001
Corticosteroids + colchicine	12 (3%)	5 (2%)	6 (4%)	1 (6%)	0	Ns
Shift NSAIDs → corticosteroids	14 (4%)	6 (3%)	6 (4%)	2 (13%)	0	Ns
No PE-targeted therapy	214 (56%)	151 (69%)	63 (43%)	3	3	<0.0001
Urgent transfer				3	3	

NSAIDs = nonsteroidal anti-inflammatory drugs; Ns = not significant.

**Table 7:** Specific data of patients transferred due to cardiac tamponade.

Age/ Sex	Type of Surgery	Surgical Approach	PS Comps	First TTE PEf Severity	First TTE PEf Site	Early PEf	Coagulation Therapy	PEf Therapy	PS Day
83/F	SAVR + ACBPG	Sternotomy	3	Mild	Loculated (right sections)	No	ASA + enoxaparin	No	18
46/M	Bentall	Sternotomy	3	Mild	Circumferential	Yes	OAC + enoxaparin	No	11
77/F	SAVR + aortic	Sternotomy	5	Mild	Circumferential	No	OAC	No	15
70/M	Tirone David	Sternotomy	4	Moderate	Loculated (left sections)	Yes	ASA + OAC	Cortisone + Colchicine (3 days)	12
82/M	SAVR + ACBPG	Sternotomy	2	Severe	Circumferential	No	ASA + OAC	Cortisone (10 days)	25
37/M	SAVR + aortic	Sternotomy	3	Severe Tamponade	Circumferential	Yes	ASA + OAC	Urgent transfer	14
80/F	ACBPG	Sternotomy	4	Severe Tamponade	Circumferential	Yes	OAC	Urgent transfer	5
77/M	CABG	Sternotomy	2	Severe Tamponade	Loculated (right sections)	No	ASA + enoxaparin	Urgent transfer	13

SAVR = surgical aortic valve replacement; ACBPG = aortocoronary bypass graft; OAC = oral anticoagulant; ASA = acetylsalicylic acid; PS = post-surgery; TTE = transthoracic echocardiogram.

## Data Availability

Data are available upon reasonable request at www.Zenodo.org.

## Abbreviations

Pericardial effusion (PEf), postpericardiotomy syndrome (PPS), cardiac tamponade (CT), European Society of Cardiology (ESC), cardiac rehabilitation (CR), transthoracic echocardiogram (TTE), nonsteroidal anti-inflammatory drugs (NSAIDs), standard deviation (SD), coronary artery bypass graft (CABG), body mass index (BMI), left ventricular ejection fraction (LVEF), heart failure (HF), postoperative atrial fibrillation (POAF), C-reactive protein (CRP), left ventricular (LV), acetylsalicic acid (ASA).
